# Unbiased compound-protein interface mapping and prediction of chemoresistance loci through forward genetics in haploid stem cells

**DOI:** 10.18632/oncotarget.24305

**Published:** 2018-01-23

**Authors:** Moritz Horn, Virginia Kroef, Kira Allmeroth, Nicole Schuller, Stephan Miethe, Martin Peifer, Josef M. Penninger, Ulrich Elling, Martin S. Denzel

**Affiliations:** ^1^ Max Planck Institute for Biology of Aging, Cologne D-50931, Germany; ^2^ CECAD-Cluster of Excellence University of Cologne, Cologne D-50931, Germany; ^3^ Center for Molecular Medicine Cologne, University of Cologne, Cologne D-50931, Germany; ^4^ Department of Translational Genomics, Center of Integrated Oncology Cologne–Bonn, Medical Faculty University of Cologne, Cologne D-50931, Germany; ^5^ Institute of Molecular Biotechnology of the Austrian Academy of Science, Vienna Biocenter, Vienna A-1030, Austria

**Keywords:** haploid stem cells, forward genetic screens, target identification, interaction site mapping, chemoresistance prediction

## Abstract

Forward genetic screens in haploid mammalian cells have recently emerged as powerful tools for the discovery and investigation of recessive traits. Use of the haploid system provides unique genetic tractability and resolution. Upon positive selection, these screens typically employ analysis of loss-of-function (LOF) alleles and are thus limited to non-essential genes. Many relevant compounds, including anti-cancer therapeutics, however, target essential genes, precluding positive selection of LOF alleles. Here, we asked whether the use of random and saturating chemical mutagenesis might enable screens that identify essential biological targets of toxic compounds. We compare and contrast chemical mutagenesis with insertional mutagenesis.

Selecting mutagenized cells with thapsigargin, an inhibitor of the essential Ca^2+^ pump SERCA2, insertional mutagenesis retrieved cell clones overexpressing SERCA2. With chemical mutagenesis, we identify six single amino acid substitutions in the known SERCA2-thapsigargin binding interface that confer drug resistance. In a second screen, we used the anti-cancer drug MG132/bortezomib (Velcade), which inhibits proteasome activity. Using chemical mutagenesis, we found 7 point mutations in the essential subunit Psmb5 that map to the bortezomib binding surface. Importantly, 4 of these had previously been identified in human tumors with acquired bortezomib resistance. Insertional mutagenesis did not identify Psmb5 in this screen, demonstrating the unique ability of chemical mutagenesis to identify relevant point mutations in essential genes.

Thus, chemical mutagenesis in haploid embryonic stem cells can define the interaction of toxic small molecules with essential proteins at amino acid resolution, fully mapping small molecule-protein binding interfaces.

## INTRODUCTION

Studying protein-protein and protein-small molecule interactions is of critical importance to understand fundamental biological processes and to advance drug development strategies. A detailed understanding of the interaction of chemotherapeutic compounds with their respective protein targets is critical (i) for on- and off-target toxicity studies during drug development, (ii) for target deconvolution, and (iii) for the anticipation of chemoresistance mutations in treated patients.

Traditionally, biochemical and biophysical approaches have been successfully applied to unravel such interactions and to define binding interfaces [1]. However, most of these strategies require previous knowledge of interacting partners and thus come with a certain bias, are highly labor intensive, and can fail to resolve the specific interaction surface. We wondered whether next-generation sequencing technologies and massively parallel genetic approaches such as saturating mutagenesis could address this problem. Can an unbiased genomic approach point to physical interactions and predict chemoresistance?

Forward genetic screens using chemical mutagenesis have revealed genetic architectures in a variety of model organisms including the fruit fly *Drosophila melanogaster*, the nematode *Caenorhabditis elegans*, and budding yeast *Saccharomyces cerevisiae* [2–4]. Alkylating agents such as ethyl methansulfonate (EMS) or N-ethyl-N-nitrosourea (ENU) are used to induce single nucleotide variants (SNVs) [5]. This allows for the investigation of a broad range of functional consequences including loss-of-function, partial loss-of-function or separation-of-function, and gain-of-function mutations [6]. Moreover, such screens are not limited to non-essential genes, but cover the entire genome and the obtained point mutations can give insights into structure-function relationships. In mammalian systems, however, these powerful genetic approaches are limited since newly induced mutations remain heterozygous, frequently resulting in a masked phenotype due to the remaining functional allele. Still, mutagenesis screens have been applied to both mice and mouse embryonic stem cells recovering dominant phenotypes [7, 8]. Moreover, in mammalian cells a variety of other forward genetic approaches emerged that have been highly successful: Knockdown screens with RNA interference libraries [9, 10], knockout screens using small guide RNA libraries with the recent CRISPR/Cas9 technology [11], and knockout strategies in haploid mouse stem cells or near-haploid human cell lines using insertional mutagenesis [12]. Such approaches not only unraveled fundamental biological principles, but also confer essential tools for drug target identification and deconvolution in pharmaceutical development. However, all knockout approaches are limited to genetic loss-of-function and largely fail to address mutations in the almost 2000 essential genes [13]. Importantly, all of these approaches cannot resolve functional changes at the amino acid level and thus preclude structure-function analysis.

Recent publications demonstrate that the combination of mouse haploid embryonic stem cells with chemical mutagenesis comprises a powerful tool to transfer the benefits of classical mutagenesis screens from lower organisms to a mammalian system [14, 15]. Given the complete coverage and the high potential resolution of this approach we asked if saturating chemical mutagenesis could uncover physical interactions and predict chemoresistance mutations. As a benchmark, we used insertional mutagenesis in parallel.

Using two toxic compounds, thapsigargin and the anti-cancer drug MG132/bortezomib, we here show that a chemical mutagenesis screen in haploid stem cells uncovers suppressor mutations in essential genes and predicts chemoresistance loci. Importantly, this genetic screening approach enables mapping of compound-target interaction surfaces at amino acid resolution.

## RESULTS

### Random insertional mutagenesis shows that Atp2a2/SERCA2 overexpression suppresses thapsigargin toxicity

**Figure 1:**
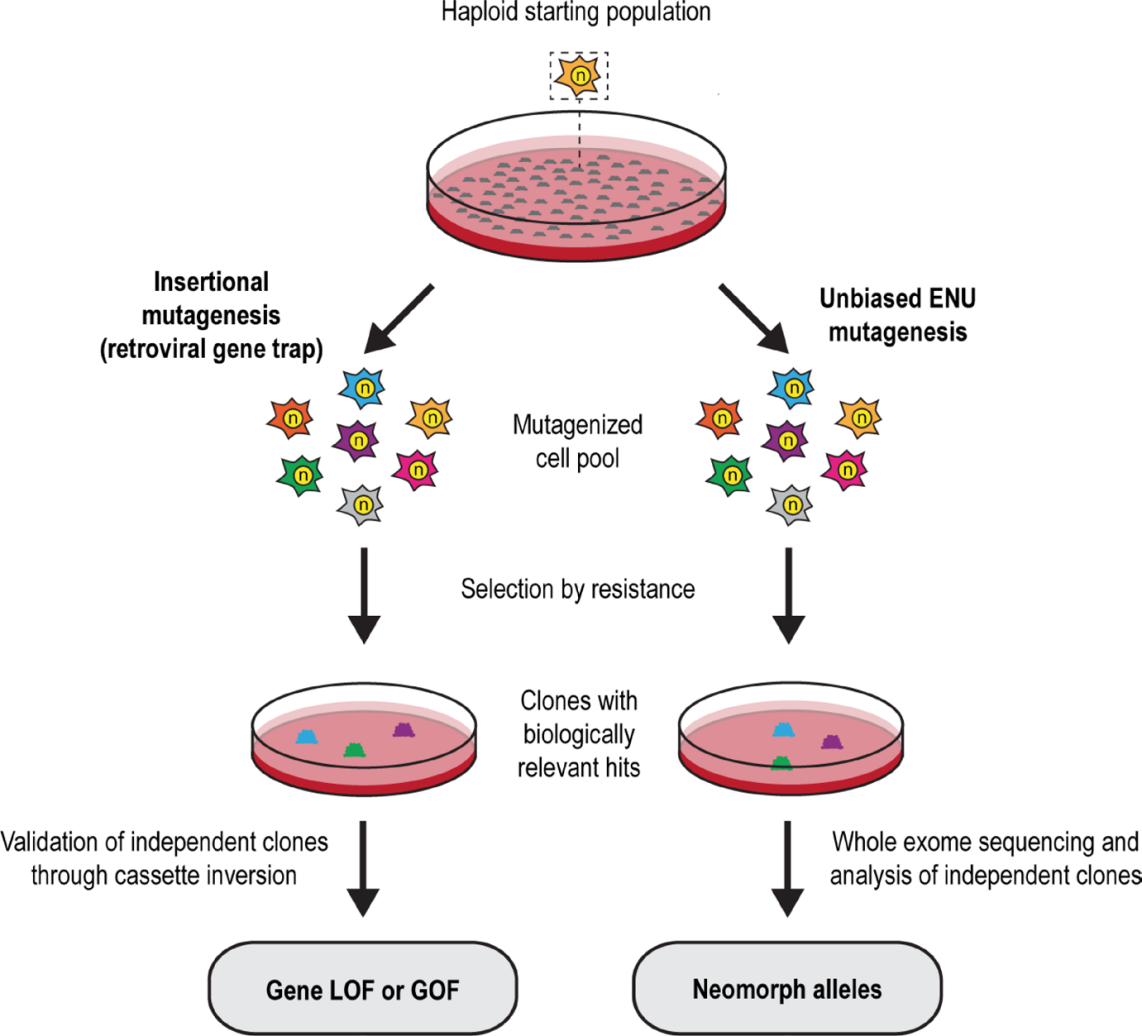
Insertional and ENU-based mutagenesis in mouse haploid embryonic stem cells Schematic representation of experimental workflow for both screening approaches. LOF loss-of-function; GOF gain-of-function.

In parallel approaches we compared and contrasted insertional mutagenesis using an enhanced gene trapping system [16] with unbiased chemical mutagenesis in mouse haploid embryonic stem cells (Figure 1). We asked whether ENU-based mutagenesis combined with next generation sequencing can give deeper insights into structure-function relations and chemoresistance mechanisms compared to classical gene trap-based approaches. Since we were particularly interested in essential processes we used the toxic drug thapsigargin to establish the screens.

**Figure 2:**
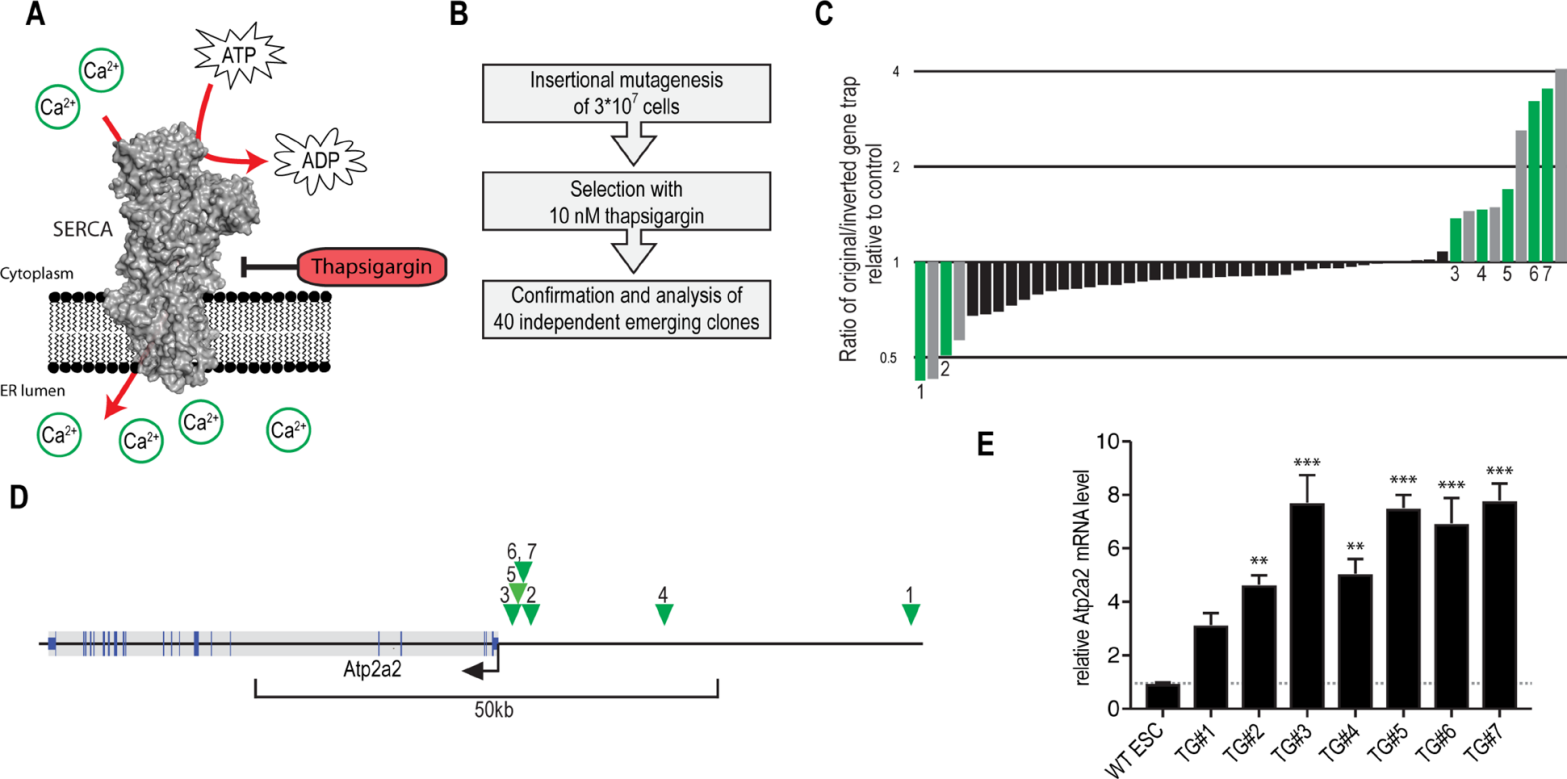
Thapsigargin-resistance screen using insertional mutagenesis validates Atp2a2/SERCA2 overexpression as resistance mechanism (**A**) Schematic representation of the SERCA2 Ca^2+^-ATPase. (**B**) Schematic representation of the experimental workflow for thapsigargin-resistance screen using insertional mutagenesis. Confirmation and analysis includes assessment of resistance dependence on insertion orientation followed by insertion site mapping. (**C**) Analysis of thapsigargin resistance with respect to the gene trapping cassette orientation. Green and grey bars indicate clones in which the gene trapping positions affected thapsigargin resistance. Green bars indicate clones in which genomic insertion sites were successfully mapped. (**D**) Schematic representation of genomic integration sites from thapsigargin-resistant colonies (green triangles). Only clones with thapsigargin resistance linked to the trapping cassette orientation were analyzed by inverse PCR. (**E**) qRT-PCR analysis of thapsigargin-resistant clones with gene trapping insertions upstream of the Atp2a2 gene. Relative Atp2a2 mRNA levels from ≥3 repeats (Mean ± SEM) are shown. ^***^p < 0.001, ^**^p < 0.01 (ANOVA).

Thapsigargin, a sequiterpene lactone isolated from the mediterranean plant *Thapsia garganica*, is a potent inhibitor of the essential endoplasmic/sarcoplasmic reticulum Ca^2+^ transport ATPase SERCA2, which is encoded by the Atp2a2 gene [17, 18] (Figure 2A). SERCA2 inhibition perturbs Ca^2+^ homeostasis causing ER-stress and apoptotic cell death [19]. Therefore, it is studied as a prodrug in a number of proliferative diseases, in particular prostate cancer and hepatocellular carcinomas [20]. Since the concrete mechanism of SERCA2 inhibition is well understood we used it as a proof-of-concept compound to assess the utility of both screening approaches [17, 21, 22].

Using a high complexity gene-trapping library we mutagenized more than 30 million mouse embryonic stem cells and screened for resistance to a toxic dose of thapsigargin (Figure 2B). 40 independent resistant colonies emerged, which were analyzed by Cre-mediated inversion of the gene-trapping cassette. We did not identify a strong resistance bias for either direction of the gene cassette, which is untypical for gene-trapping screens [23] Figure 2C). Moreover, resistance further increased in some cases upon inversion of the splice acceptor, while inversion to the putatively non-disruptive antisense orientation is predicted to revert phenotypes. To understand this phenomenon, we mapped multiple gene trap insertion sites using inverse PCR. Interestingly, in all analyzed clones, resistance-linked cassette insertions mapped to the promoter region of the Atp2a2 gene (Figure 2D). In agreement with the design of the enhanced gene-trapping cassette (that contains an array of Oct4 binding sites [16]) these integrations led to a strong increase in Atp2a2/SERCA2 expression (Figure 2E). Hence, insertional mutagenesis confirmed that Atp2a2/SERCA2 overexpression counters thapsigargin toxicity, a resistance mechanism, that was previously described [24]. Interestingly, none of the analyzed insertions mapped to Atp2a2 introns or exons, indicating that no LOF alteration resulted in thapsigargin resistance.

### Chemical mutagenesis separates functions of the thapsigargin target Atp2a2/SERCA2 and defines the binding interface at amino acid resolution

In a next step, we established a chemical mutagenesis approach in haploid cells to assess whether we can separate functions in an essential gene to uncover additional thapsigargin toxicity suppressors.

First, we defined the minimal mutagenic treatment in haploid mouse embryonic stem cells using the toxin 6-thioguanine (Supplementary Figure 1). Inactivation of the hypoxanthine-guanine phosphoribosyltransferase (*Hprt1*) gene and mutations in a number of DNA mismatch repair genes lead to 6-thioguanine resistance [25, 26]. This limited and well-defined set of resistance loci allows for precise titration of the mutagen. In order to avoid high mutation loads for subsequent genomic analyses, we selected the lowest ENU dose that increased the number of 6-thioguanine resistant colonies over spontaneous resistance in non-mutagenized cells (0.01 mg/ml).

**Figure 3:**
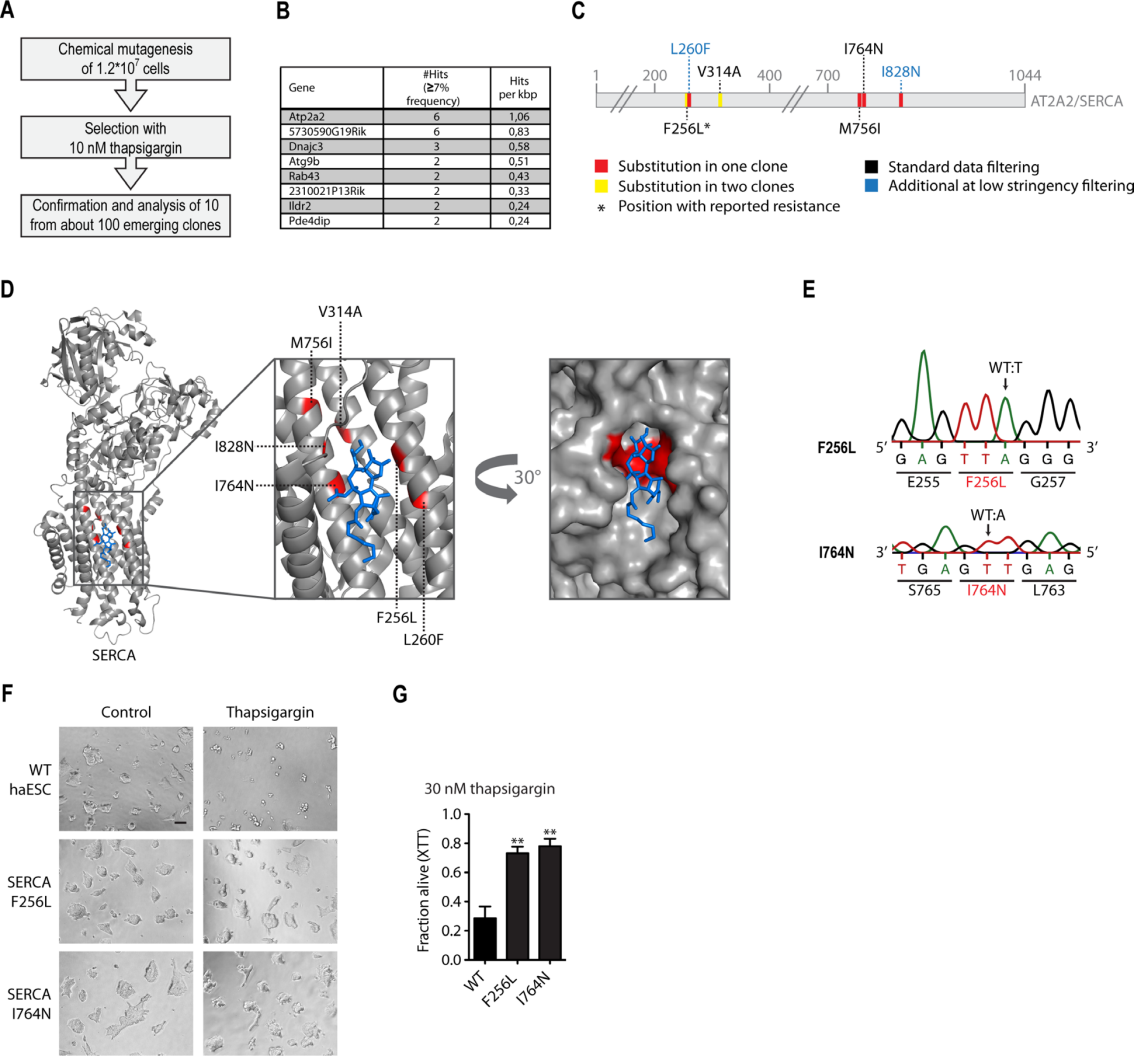
Chemical mutagenesis screen for thapsigargin resistance reveals amino acid substitutions in Atp2a2/SERCA2 (**A**) Schematic representation of the experimental workflow for thapsigargin-resistance screen using chemical mutagenesis. (**B**) Table listing candidate suppressor genes for thapsigargin-resistance identified by whole exome sequencing. Genes are sorted by the number of hits recovered after standard filtering (≥7% mutant allele frequency) per kbp. (**C**) Schematic representation of the SERCA amino acid sequence. Substitutions inferred from whole exome sequencing analysis with low stringency (≥3% mutant allele frequency) and their positions are highlighted. (**D**) Structure of rabbit SERCA2 (grey) in complex with thapsigargin (blue). Identified substitutions are highlighted in red. *pdb:5a3q*. (**E**) Genotyping (Sanger sequencing) of CRISPR/Cas9 engineered Atp2a2/SERCA2 alterations and their consequences at the amino acid level. (**F**) Representative images of WT cells and two engineered Atp2a2/SERCA2 suppressor candidates after 48 hours of treatment with 30 nM thapsigargin or respective control. Scale bar, 100 µm. (**G**) Cell viability assay (XTT) of cells with the Atp2a2/SERCA2 thapsigargin-suppressor candidate mutations engineered by CRISPR/Cas9 genome editing, and unaltered WT controls. ^**^p < 0.01 (ANOVA). Mean ± SEM (*n* = 3).

Then we performed a thapsigargin resistance ENU mutagenesis screen. After mutagenesis, a pool of 12 million haploid cells was subjected to selection with 10 nM thapsigargin for 21 days, resulting in the growth of 97 resistant colonies (Figure 3A). We confirmed thapsigarginresistance using the XTT viability assay in a random selection of the emerging clones, showing significant resistance in all cases (Supplementary Figure 2A). To exclude increased Atp2a2/SERCA2 expression as a resistance mechanism, we measured Atp2a2 mRNA levels by qRT-PCR in the selected clones (Supplementary Figure 2B). None of the analyzed clones showed relevant changes in Atp2a2 expression.

In order to identify causative ENU-induced gene mutations, we performed next-generation whole exome sequencing. We reasoned that independent mutations would be overrepresented in a causative gene. Thus, our unbiased approach requires sequence analysis of multiple resistant clones, which led us to sequence pooled genomic DNA of all 10 resistant clones (Supplementary Figure 2C). This approach reduces the number of exome library preparations and allows parallel analysis of multiple resistant clones. Using this approach it would be possible to directly identify causative mutations in a resistance gene.

Next-generation whole exome sequencing was performed at deep coverage (150×). Given the experimental design, an individual SNV from one of the clones was expected to contribute an average of 10% of the mutant allele frequency in the exome sequence analysis (Supplementary Figure 2C).

Data analysis was performed using a cut-off at ≥7% mutant allele frequency. This resulted in a list of 8 candidate genes that carried at least two mutations in their coding regions that affect protein function (missense, frame shift, splice variants, or premature stops). Candidates were ranked by normalizing the number of identified mutations within a gene to its coding sequence size. This analysis revealed Atp2a2/SERCA2 as the top candidate (Figure 3B). Importantly, a fully independent bioinformatic approach confirmed our ranking. The latter pipeline was developed for complex samples from human cancers with multiple contributing clonal populations and is based on a probabilistic model instead of clear cut-offs [27–29]. Despite the fact that no filters regarding the mutation consequence were applied, the resulting ranking was even clearer (Supplementary Table 1). Atp2a2 emerged as the top candidate while all other candidate genes in the list only harbored silent mutations.

The initial standard filtering approach identified 5 independent Atp2a2 mutations, of which one, V314A, was present in two clones as judged by the mutant allele frequency of 22,6% (Figure 3C and Supplementary Table 2). Closer inspection of the raw sequencing data revealed two additional Atp2a2 mutations that had not passed the stringent data processing (Figure 3C and Supplementary Table 2). Thus, a total of 8 Atp2a2 SNVs resulting in amino acid substitutions emerged from the ten analyzed clones. Possibly, the two remaining clones acquired thapsigargin resistance through a different mechanism. Interestingly, two clones harbored independent mutations resulting in the F256L substitution, a variant previously reported to confer thapsigargin-resistance in mammalian cells [30]. This result supports the notion that true resistance-conferring alleles were identified through our unbiased whole-genome approach.

Given the high number of identified substitutions across the SERCA2 amino acid sequence we wondered whether they all confered resistance and if this approach might provide structural information regarding the interaction between thapsigargin and SERCA2. We highlighted the substitutions in a recent crystal structure of *Oryctolagus cuniculus* SERCA2 with thapsigargin [31] (Figure 3D). Strikingly, all identified Atp2a2/SERCA2 mutations map to the thapsigargin binding site or are in close proximity within the tertiary structure. Thus, sequencing of only ten resistant clones allowed prediction of the thapsigargin-SERCA interaction surface. Only minor side chain alterations would be predicted to abrogate thapsigargin binding, particularly at positions F256 and I764 (Figure 3D). Next, we aimed to validate two mutations by genetic engineering of WT haploid stem cells. The CRISPR/Cas9 system was used to introduce the F256L and the I764N mutation, respectively, into the Atp2a2 locus. Through direct thapsigargin selection of a cell population after transfection with the targeting CRISPR/Cas9 mix we confirmed causality of these mutations in thapsigargin resistance (Supplementary Figure 2D and 2E). Sanger sequencing validated the substitutions in the resistant CRISPR-engineered clones (Figure 3E). Both SERCA substitutions, F256L and I764N, resulted in strong thapsigargin resistance, as assayed in the XTT viability assay (Figure 3F and 3G). We thus confirm the reported F256 resistance locus and identify additional amino acid substitutions in the thapsigargin binding pocket that confer drug resistance.

### Insertional mutagenesis fails to identify MG132/bortezomib resistance mechanisms

**Figure 4:**
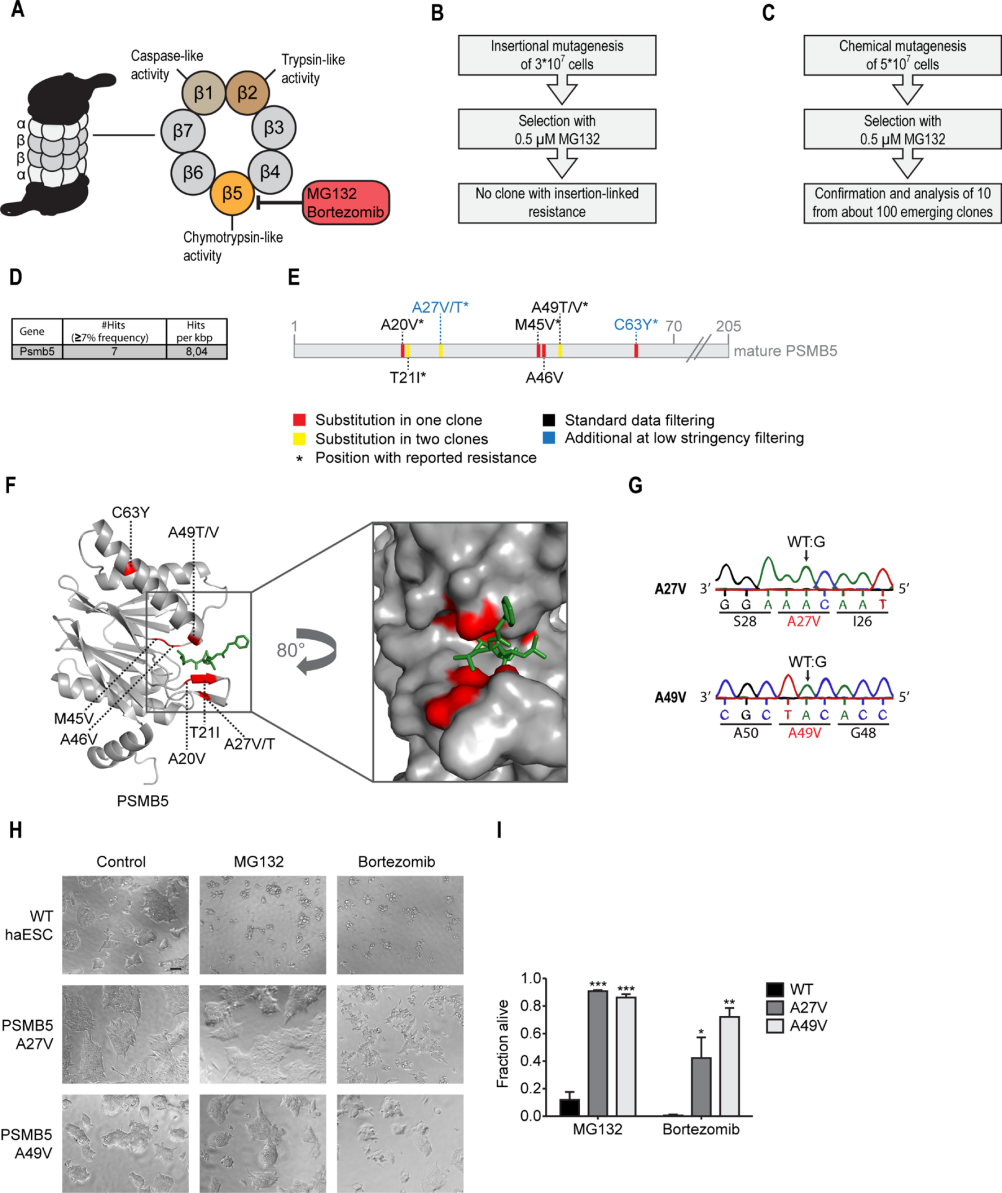
MG132 resistance screen using chemical mutagenesis identifies suppressor mutations in PSMB5 and maps the PSMB5-MG132 binding interface (**A**) Schematic representation of proteasome inhibitors MG132 and bortezomib targeting the β5 subunit (PSMB5). (**B**) Schematic representation of the experimental workflow for MG132/bortezomib-resistance screening using insertional mutagenesis. No clone showed MG132 resistance linked to the gene trapping insertion. (**C**) Schematic representation of the experimental workflow for MG132/bortezomib-resistance screening using chemical mutagenesis. (**D**) Table showing the only candidate suppressor gene for MG132-resistance identified by whole exome sequencing. The number of hits recovered after standard filtering (≥7% mutant allele frequency) per kbp is displayed. (**E**) Schematic representation of the PSMB5 amino acid sequence. Substitutions inferred from whole exome sequencing analysis with low stringency (≥3% mutant allele frequency) and their positions are highlighted. (**F**) Structure of yeast PSMB5 (grey) in complex with MG132 (green). Identified substitutions are highlighted in red. *pdb:5d0t.* (**G**) Genotyping (Sanger sequencing) of CRISPR/Cas9 engineered Psmb5 candidate mutations and their consequences at the amino acid level. (**H**) Representative images of WT cells and cells carrying two engineered Psmb5 suppressor candidate mutations after a 48 hours treatment with 0.5 µM MG132, 50 nM bortezomib, or respective control. Scale bar, 100 µm. (**I**) Cell viability assay (XTT) of Psmb5 MG132-suppressor candidates engineered by CRISPR/Cas9 genome editing and WT controls. ^*^p < 0.05, ^**^p < 0.01, ^***^p < 0.001 (ANOVA). Mean ± SEM (*n* = 3).

Given the encouraging results from the thapsigargin resistance screen we asked whether our screening pipeline could be applied to a relevant anti-cancer drug. We decided to assess resistance to MG132, which was the lead compound in the development of bortezomib, a proteasome inhibitor used in the treatment of multiple myeloma and mantle cell lymphomas [32, 33]. MG132 and bortezomib inhibit the proteasome by reversible interaction with the catalytic β5 subunit (PSMB5), which harbors the chymotrypsin-like-proteolytic activity and is therefore essential for cell survival (Figure 4A) [34, 35]. Resistance to bortezomib is common in relapsed multiple myeloma patients and can be caused by point mutations in the bortezomib binding site of PSMB5 or by *PSMB5* overexpression [36]. We thus asked whether our forward mutagenesis approaches in haploid stem cells might have the capacity to predict these resistance mechanisms and to give detailed insights into the drug’s mode of action.

First, we applied MG132 selection to the insertional mutagenesis approach (Figure 4B). From about 30 million mutagenized cells, only 9 independent MG132-resistant colonies emerged. Surprisingly, the resistance of all colonies was independent from the gene trapping cassette orientation suggesting no causality between insertion site and resistance mechanism. Such clones had potentially acquired spontaneous genetic alterations independent of the inserted cassette. Thus, corresponding gene trap integration sites were not further analyzed.

### Chemical mutagenesis identifies clinically relevant chemoresistance alleles and maps the MG132-PSMB5 binding interface

Interestingly, a recent targeted approach has recovered multiple bortezomib resistance substitutions in the *PSMB5* locus. A modified CRISPR/Cas9 system that recruits the somatic hypermutation machinery to a small target space was used to introduce SNVs [37]. This genetic approach required previous knowledge about the compound’s target. Thus, we asked whether our chemical mutagenesis approach could predict the resistance gene *de novo* from the entire genome.

We generated a mutagenized cell pool of 50 million haploid cells and selected with 0.5 µM MG132 for 21 days (Figure 4C). From 105 resistant colonies, we confirmed MG132-resistance in 10 randomly selected haploid clones (Supplementary Figure 3A). Since proteasome activation (e.g. through overexpression) can overcome proteasome inhibitory effects [38], we assessed proteasome activity of the 10 MG132 resistant clones. Interestingly, the chymotrypsin-like proteasome activity was reduced in all clones, while caspase-like activity remained largely intact (Supplementary Figure 3B and 3C). About half of the clones (13, 16, 45, 46, and 51) where partially refractory to MG132 treatment, while WT cells showed a 94% reduction in chymotrypsin-like proteasome activity (Supplementary Figure 3B). Together, these data indicate that ENU-induced MG132 resistance mutations might directly affect the proteasome. To test this, we performed whole exome sequencing of a pooled DNA library from the 10 selected clones. Applying our bioinformatics filtering analysis retrieved a single candidate gene, Psmb5 (Figure 4D). Again, the independent cancer genomics bioinformatic approach confirmed Psmb5 as the top candidate (Supplementary Table 3). We identified 6 independent mutations in Psmb5. One of these, resulting in the T21I substitution, was present in two clones as judged by the mutant allele frequency of 18,6% (Figure 4E and Supplementary Table 4). Closer inspection of the initial data revealed that three additional Psmb5 mutations did not pass the stringent data processing (Figure 4E and Supplementary Table 4). Thus, all 10 clones analyzed harbored Psmb5 mutations. Given the longtime use of bortezomib treatment in cancer patients, a number of resistance mechanisms are known [39]. Most prominently, Psmb5 mutations or overexpression suppress bortezomib toxicity [40]. Multiple SNVs from our screen resulted in identical changes, as reported in these previous studies (Figure 4E). This indicates causality for MG132 resistance and underscores the ability of the mutagenesis pipeline to predict such clinically relevant alterations. To further test the capacity of our genetic approach to map biochemical interaction sites, we highlighted all identified amino acid substitutions in a crystal structure of the yeast proteasome bound to MG132 (Figure 4F) [41]. Strikingly, all changes map to the PSMB5-MG132 interaction surface, with exception of the C63Y substitution, which was previously reported to induce conformational alterations of the subunit [42]. These findings point to a critical role for all identified substitutions in MG132 and bortezomib resistance. To further validate the results of the unbiased screen, two resistance mutations were selected and engineered in WT haploid stem cells using CRISPR/Cas9-mediated genome editing (Figure 4G). While the A49V substitution was previously reported to confer resistance in multiple cellular systems [36, 43], the A27V alteration was just recently found in a bortezomib resistant myeloma patient [44]. Both individually engineered Psmb5 mutations conferred strong resistance to MG132, bortezomib and the related proteasome inhibitor carfilzomib (Figure 4H and 4I, Supplementary Figure 3D).

Thus, largely unbiased chemical whole genome mutagenesis separates PSMB5 functions and uncovers MG132/bortezomib interactions without any previous knowledge regarding a compound’s target. These observations validate the screening pipeline as an unbiased tool to identify genetic resistance loci and to map specific compound-protein interaction sites at amino acid resolution.

## DISCUSSION

In this study, we compared insertional and chemical mutagenesis approaches in mouse haploid embryonic stem cells in two independent screens for thapsigargin and MG132/bortezomib resistance. Insertional mutagenesis resulted in thapsigargin resistance through overexpression of the direct target Atp2a2/SERCA2, but failed to induce resistance mechanisms in the MG132 screen. Chemical mutagenesis combined with next generation sequencing revealed resistance mutations in the respective drug targets Atp2a2/SERCA2 and Psmb5/PSMB5. Genome engineering in haploid stem cells validated these specific individual amino acid substitutions. Through random ENU-mutagenesis and saturation screening, the pipeline recovered functionally relevant alleles that confer drug resistance in cellular systems and cancer patients. Exome sequencing analysis resolved large parts of the compound-protein interaction surface for both drugs. This demonstrates that unbiased forward genetic screens using chemical mutagenesis in haploid stem cells is highly complementary to currently applied gene trapping strategies and can enhance the screening resolution to allow structure-function analyses and interaction site mapping.

The discovery of near-haploid and fully haploid mammalian systems has revolutionized genetic screening options. By applying a variety of insertional-based mutagenesis approaches, forward genetics in haploid cells uncovered multiple modes of drug action as well as fundamental biological principles. Here, we extend the insertional approach to gain-of-function resistance mechanisms such as the overexpression of Atp2a2/SERCA2. Overexpression of the drug target gene was not identified in the MG132-resistance screen, potentially because the direct target PSMB5 is part of the multi-subunit proteasome, and increased amounts of only one subunit might not suffice to induce resistance. Moreover, limitations of the insertional mutagenesis approach also become evident as the mutagenic splice acceptor cassettes largely induce gene loss-of-function, precluding analysis of essential genes. Therefore, no specific alterations in the essential drug targets Atp2a2/SERCA2 and Psmb5*/*PSMB5 could be identified. In contrast to insertional mutagenesis, chemical mutagenesis in haploid cells covers the entire genomic landscape and recovers not only loss-of-function, but also separation- or partial loss-of-function, as well as gain-of-function mutations of candidate genes. The only limitation is the mutagenesis bias of a given mutagen. In contrast to knockout strategies such as insertional mutagenesis- or CRISPR-Cas9-based screens, this approach detects relevant amino acid substitutions in essential genes as demonstrated for Atp2a2/SERCA and Psmb5.

Selecting for MG132 resistance following chemical mutagenesis, we retrieved specific mutations conferring resistance to MG132, the lead compound of the approved chemotherapeutic compound bortezomib (clinical name “Velcade”). Identical substitutions have been reported in numerous bortezomib-resistant cancer cell lines and were also identified in a patient resistant to bortezomib therapy [45]. Conceivably, forward mutagenesis screens in haploid stem cells could be highly beneficial for chemotherapeutic drug development: modeling the selection pressure *in cellulo* can predict potential genetic resistance mechanisms that otherwise would manifest in patients only years later. This predictive capacity therefore has potential to improve specificity in early drug development.

Intriguingly, the unbiased screening approach in haploid cells not only revealed protein targets of drug toxicity, but also provided detailed information of the compound-target protein interaction surface. Analysis of only 10 clones was sufficient to achieve this. For both thapsigargin and MG132/bortezomib, identified suppressor mutations plausibly interfere with the physical drug-protein interaction. By inference, such information can reveal mechanisms of action, i.e. for drug candidates from phenotypic screens, which is currently the drug development strategy resulting in most new molecular identities [46, 47]. Given the high resolution at the amino acid level, this might promote rational drug optimization. Similarly, unbiased interaction mapping could be applied to identify off-target toxicity mechanisms. This could result in rational modifications of chemical structures of potent compounds with toxic side effects.

Notably, given these potential applications, it is important to keep in mind that our unbiased haploid stem cell screening pipeline still faces numerous challenges. First, cancer biology is highly complex and oftentimes only a combination of deleterious mutations results in uncontrolled proliferation, a phenotype targeted by most anti-cancer therapies. Predicting resistance mechanisms in haploid stem cells would therefore require several genetic alterations to mimic the carcinogenic situation. Second, such complex phenotypes might also lead to a number of possible resistance mechanisms and interaction partners, which will be a challenge for suppressor candidate identification and validation. Third, certain target genes might not be expressed in the stem cells, complicating the screen. Finally, especially with regard to target deconvolution, the relevant read-out will need to be more specific than resistance to toxic compounds, necessitating gene expression or protein modification analysis. However, given the genetic tractability of the haploid system, as demonstrated by our CRISPR/Cas9-mediated target validation and the recent development of several reporter knock-ins [48], infinite numbers of tailored genetic backgrounds and a wide range of read-out strategies can be generated. Further, pluripotency of the cells allows differentiation into specific cell lineages to mimic many physiological conditions [49]. Moreover, our screening approach can rapidly be adjusted to new emerging haploid or near-haploid systems such as human embryonic stem cells [50]. Lastly, we have demonstrated that whole exome sequencing of pooled DNA libraries results in reliable candidate identification. This opens the door to address more complex genetic interactions by sequencing high numbers of candidate clones.

Taken together, chemical mutagenesis in haploid mammalian cells adds ample opportunities to benchmark forward genetic screening approaches. This highly versatile approach allows identification of drug targets and to uncover clinically relevant drug resistance mechanisms with a fast turn-around time. The combination of genome wide loss- and gain-of-function screening using insertional mutagenesis in haploid cells with neomorph alleles generated by chemical mutagenesis achieves an unmatched resolution in chemogenomics, allowing for direct mapping of compound-target interaction surfaces at amino acid level. In the future, this screening paradigm might contribute to drug development, on- and off-target toxicity studies, as well as to individualized cancer therapies.

## MATERIALS AND METHODS

### Cell lines and culture conditions

AN3-12 mouse embryonic haploid stem cells were used for all experiments in this study. They were cultured as previously described [12]. In brief, DMEM high glucose (Sigma-Aldrich, St. Louis, Missouri) supplemented with glutamine, fetal bovine serum (15%), streptomycin, penicillin, non-essential amino acids, sodium pyruvate, β-mercaptoethanol and LIF was used on non-coated tissue culture plates.

### Cell sorting

To maintain a haploid cell population cells were stained with 10 µg/ml Hoechst 33342 (Thermo Fisher Scientific, Waltham, Massachusetts) for 30 min and sorted for DNA content on a FACSAria Fusion sorter (BD, Franklin Lakes, New Jersey). The haploid 1n peak was purified from a viable population (propidium iodide staining negative) and flow profiles were recorded with the FACSDiva software (BD).

### Insertional mutagenesis screening

#### Retroviral infection of ES cells

Oct4 enhanced gene trap retroviruses carrying a splice acceptor followed by a neomycin resistance gene in 3 reading frames and Oct4 binding sites to enhance transcription [16] were packaged in Platinum E cells (Cell Biolabs), concentrated by centrifugation (25,000 rpm, 4°C, 4 h) and applied to ES cells with 2 μg polybrene per ml for 8 hours. Selection for gene trap insertions was done using G418 (Gibco) at 0.2 mg/ml. To estimate numbers of integrations 500.000 cells were plated on 15 cm dishes, selected for integrations using G418 selection and colonies counted after 10 days. For comparison, 5.000 cells were plated without selection.

#### Selection of mutagenized cell populations

From 2 vials of a barcoded AN3-12 Retro Library (68 million cells per vial, 77 million complexity) 3 million cells per plate were seeded to 20 × 15 cm plates. Half of the plates were selected with 10 nM thapsigargin, the other half with 0.5 µM MG132. Plates were fed with medium containing the respective drug daily until day 7 post drug treatment start. At day 9, plates were fed with medium without drugs and at day 11, plates were fed with medium containing 2× LiF and the respective drug. At day 14 post drug treatment start, emerging colonies were picked.

For genomic DNA isolation, cells from confluent 24-wells were lysed by washing with PBS, dry freezing and overnight lysis in Genomic DNA Lysis Buffer (10 mM Tris-HCl pH 8.0, 5 mM EDTA, 100 mM NaCl, 1% SDS, 1 mg/mL Proteinase K). gDNA was then precipitated with Isopropanol, washed with 70% Ethanol and solved in TE.

#### Alamar blue assay

In a 24-well plate clones were treated with their respective drug (concentration as in the screen). Additionally, wildtype AN3-12 cells were kept the same way in three different densities. After 48 hours, clones and AN3-12 were split 1:10 and kept in drug treatment. 96 hrs after treatment start, cell density was measured with resazurin (140 µM, Sigma, R7017-1G) on a Synergy Multi-Mode Reader (BioTek).

#### Barcode PCR

To validate independence of the resistant clones a Barcode PCR was performed on clone gDNA using the primers “barcodePCR-F” (GGTTGATCTGAGCTACTCATCAACGGT) and “barcodePCR-R” (CAAGTTCCTTCTGGTTCTGGCTCTGCT). The PCR reaction was analyzed on an agarose gel, purified with illustra ExoProStar 1-step kit (GE Healthcare, US77720) and the barcode was retrieved by Sanger Sequencing with “barcodePCR-R” primer. Sequences were analyzed with 4Peaks.

#### Red-green assay

Clones were split in a 1:5 ratio in two 24-well plates and infected with retroviral MLP-mCherry-Cre-puro and MLP-GFP-puro, respectively. 24 hours post infection Puromycin-selection (1 µg/mL) was started. Cells were split once during selection (5 days) and an aliquot was frozen.

For each clone mCherry-Cre and GFP cells were mixed in a 2:1 ratio (approximately) and seeded onto 2 independent 24-well plates. To one plate the drugs were added (same concentration as for the screen) to the other media only was added. 72 hours post seeding/drug addition, cells were FACS analysed (BD Fortessa with HTS).

#### Inverse PCR

MseI and NlaIII (NEB, R0525L and R0125L) were used in parallel to fragment the genome. After a ring ligation step (T4 DNA ligase and buffer, Roche, 10716359001) gDNA was linearized using SbfI (NEB, R0642L). The genomic region was amplified using the primers “DS” (GAGCCAGAACCAGAAGGAACTTGAC) and “US” (GTGACTGGAGTTCAGACGTGTGCTCTTC). The PCR reaction was analyzed on an agarose gel, purified by Exostar or QIAquick Gel Extraction Kit (Qiagen, 28704) and used for Sanger Sequencing with primer “DS”. Sequences were analyzed using 4Peaks, Seqman Pro and USCS Genome Browser.

### Chemical mutagenesis screening

#### Ethylnitrosourea (ENU) treatment and drug selection

For chemical mutagenesis cells were singled with Trypsin/EDTA (Thermo Fisher Scientific, Waltham, Massachusetts) and transferred to a 15 ml tube. Mutagenesis was performed in full medium for two hours at room temperature under agitation with the indicated ENU concentration. Then cells were washed with medium without LIF 5× before being transferred to a culture dish. Drug selection was performed for 21 days starting 48 hours post mutagenesis using 0.2 µg/ml 6-thiogunanine, 10 nM thapsigargin, or 0.5 µM MG132 (all Sigma Aldrich), respectively.

#### Cell viability assay (XTT)

Relative cell viability was assessed using the XTT cell proliferation Kit II (Roche Diagnostics, Basel, Switzerland) according to the manufacturer’s instructions. Drug treatments were performed for 48 hours, starting 24 hours after cell seeding. The following compound concentrations were used: thapsigargin, 30 nM; MG132, 0.5 µM; bortezomib, 50 nM; carfilzomib, 10, 25, and 50 nM. XTT turnover was normalized to corresponding untreated control cells.

#### DNA extraction and exome sequencing

DNA extraction was performed from about 1 × 10^6^ cells using the Gentra Puregene Tissue Kit (Qiagen, Venlo, Netherlands) including RNAse treatment. DNA integrity was controlled by agarose gel electrophoresis prior to exome preparation from 200 ng of pooled DNA from 10 suppressor clones. Exome-enriched libraries for Illumina paired-end multiplexed sequencing were generated using the Agilent SureSelect^XT^ mouse all exon kit following the manufacturer’s recommendation on the automated Agilent Bravo liquid handling platform. After validation (2200 TapeStation, Agilent Technologies, Santa Clara, California) and quantification (Qubit System, Thermo Fisher Scientific) pools of libraries were generated and quantified using the KAPA Library Quantification Kit (Peqlab, Erlangen, Germany) and the 7900HT Sequence Detection System (Applied Biosystems, Foster City, California). Subsequent sequencing was performed on an Illumina HiSeq4000 sequencing instrument using a paired-end 2 × 75 bp protocol.

#### Exome sequencing analysis

Raw reads were aligned to reference genome (mm9) with bwa (v.0.7.15). Reads were converted to bam format with samtools (v.1.3.1) and sorted with Picard (2.8.1). After marking duplicates with Picard, read groups were replaced with Picard AddOrReplaceReadGroups, bam/sam file reordered with Picard ReorderSam and indexed with BuildBamIndex. GATK (v.3.4.46) RealignerTargetCreator was then used to generate a list of positions for GATK IndelRealigner. After indexing, base recalibration was perform with GATK BaseRecalibrator and recalibrate reads printed with GATK PrintReads. Samtools pileup was then used to identify variants in the respective samples. After discarding indels, variants found in a control sample of 10 pooled clones that underwent mutagenesis but no selection were discarded in treated samples. Regions with a minimum read depth of 50 and where the most prominent allele was above 3% were further analyzed. After variant annotation with snpEff (v.4.2) variants relating to a moderate or high effect in protein coding were kept.

For the second approach, we aligned all reads to the reference genome (mm10) by bwa mem (version 0.7.13-r1126) and analyzed the aligned data using our in-house developed cancer analysis pipeline, as described previously [27–29]. In brief, our approach is based on a probabilistic model to call mutations and automatically subtract detected mutations from an untreated control. To call mutations, we consider coverage, allelic frequency, and forward-reverse-bias of the variant, as well as an estimation of the global sequencing error. Furthermore, our mutation calling is optimized to accurately determine mutations even with low allelic fractions. Prior to mutation detection our pipeline automatically masks duplicated reads and corrects for overlapping read pairs. All detected mutations are subsequently annotated by the pipeline’s own annotation module. Finally, only recurrently mutated genes are considered as candidates conferring drug resistance.

### Structure models

PSMB5 (accession number: 5d0t) and SERCA (accession number: 5a3q) structure figures were generated using PyMOL (The PyMOL Molecular Graphics System, Version 1.5.0.5, Schroedinger, LLC).

### RNA isolation and qRT-PCR

Cells were collected in QIAzol (QIAGEN) and frozen in liquid nitrogen. Total RNA was prepared by RNeasy Mini Kit (QIAGEN) and cDNA was subsequently generated by iScript cDNA Synthesis Kit (BioRad, Hercules, California). qRT-PCR was performed with Power SYBR Green master mix (Applied Biosystems) on a ViiA 7 Real-Time PCR System (Applied Biosystems). GAPDH expression functioned as internal control.

Atp2a2 primers:

5′-AGCCTTTGTAGAGCCGTTTG-3′ (fwd), 5′-CGATACACTTTGCCCATTTCAG-3′ (rev).

### Proteasome assays

Proteasome chymotryptic and caspase activity was assayed as the rate of hydrolysis of the fluorogenic peptide suc-LLVY-AMC (Sigma Aldrich) or Z-LLE-AMC (Enzolifesciences, Farmingdale, New York), respectively. Cell extracts were prepared in 25 mM Tris HCl, pH 7.5 using sonication. Protein lysate (20 µg) was incubated with 12.5 µM suc-LLVY-AMC or Z-LLE-AMC in a total volume of 200 µl. MG132 was spiked into the reaction well at a final concentration of 0.1 µM where indicated. AMC fluorescence was measured using 355 nm excitation and 460 nm emission filters with free AMC (Sigma Aldrich) as standard every min for 30 min at 37°C.

### Gene editing and genotyping by Sanger sequencing

Specific Atp2a2 and Psmb5 mutations were engineered in WT haploid embryonic stem cells using the CRISPR/Cas9 technology as previously described [51]. DNA template sequences for small guide RNAs were designed online (http://crispor.org), purchased from Sigma and cloned into the Cas9-GFP expressing plasmid PX458 (addgene #48138, Supplementary Table 5). Corresponding guide and Cas9 expressing plasmids were cotransfected with the respective single stranded DNA repair template (Integrated DNA technologies, Coralville, Iowa, Supplementary Table 5) using lipofectamine 3000 (Thermo Fisher Scientific) according to manufacturer’s instructions. Transfected haploid stem cells were transferred to 10 cm plates 24 hours post transfection and selected with 10 nM thapsigargin or 0.5 µM MG132, respectively, for 14 days. Resistant colonies were transferred to 24-well plates and subjected to genotyping. DNA was extracted (DNA extraction solution, Epicentre Biotechnologies, Madison, Wisconsin) and edited regions were specifically amplified by PCR (Supplementary Table 5). Sanger sequencing was performed at Eurofins Genomics GmbH, Ebersberg, Germany.

## SUPPLEMENTARY MATERIALS FIGURES AND TABLES


